# Tuberculosis of the Common Bile Duct

**DOI:** 10.1155/1991/42843

**Published:** 1991

**Authors:** Samrerng Ratanarapee, Arun Pausawasdi

**Affiliations:** 1 Department of Pathology Faculty of Medicine Siriraj Hospital Mahidol University Bangkok 10700 Thailand; 2 Department of Surgery Faculty of Medicine Siriraj Hospital Mahidol University Bangkok 10700 Thailand

## Abstract

The intrahepatic biliary tree can occasionally be infected by *Mycobacteriurn tuberculosis*, but tuberculosis of the common bile duct has not previously been reported. A 38-year-old man with obstructive jaundice, who was originally thought to have cholangiocarcinoma associated with opisthorchiasis (a common combination in Thailand), was finally proved to have tuberculosis of the common bile duct with adjacent tuberculous lymphadenitis. Following T-tube drainage and antitubercular therapy, he made a complete recovery. The importance of a tissue diagnosis in all cases of obstructive jaundice is emphasized to avoid missing rare but curable diseases.


*HPB Surgery*, 1991, Vol. 3, pp. 205-208
Reprints available directly from the publisher
Photocopying permitted by license only

© 1991 Harwood Academic Publishers GmbH
Printed in the United Kingdom

### CASE REPORT

### TUBERCULOSIS OF THE COMMON BILE DUCT

SAMRERNG RATANARAPEE and ARUN PAUSAWASDI*
*Department of Pathology and *Department of Surgery, Faculty of Medicine, Siriraj Hospital, Mahidol University, Bangkok 10700, Thailand.*

*(Received 2 July 1990)*

The intrahepatic biliary tree can occasionally be infected by *Mycobacterium tuberculosis*, but tuberculosis of the common bile duct has not previously been reported. A 38-year-old man with obstructive jaundice, who was originally thought to have cholangiocarcinoma associated with opisthorchiasis (a common combination in Thailand), was finally proved to have tuberculosis of the common bile duct with adjacent tuberculous lymphadenitis. Following T-tube drainage and antitubercular therapy, he made a complete recovery. The importance of a tissue diagnosis in all cases of obstructive jaundice is emphasized to avoid missing rare but curable diseases.

KEY WORDS: Tuberculosis, granuloma, common bile duct

### INTRODUCTION

In several countries including Thailand, tuberculosis remains one of the commonest infectious diseases[1]. Lung, bone, intestine or genitourinary tract are usually affected, but involvement of rare organs such as the thyroid and myocardium has been reported from this hospital[2,3]. We now describe a case of tuberculosis of the common bile duct, for which we can find no precedent in the literature.

### CASE REPORT

A 38-year-old male farmer from Prachinburi in the east of Thailand was admitted with a two-month history of painless obstructive jaundice. On examination he was cachectic and deeply icteric, with a normal temperature and an impalpable liver. He was anaemic (haematocrit 30 per cent). Total and differential white cell counts were normal. There were no parasitic ova in the stool. Total serum bilirubin was grossly elevated (510 umol/L), but liver enzymes were normal apart from a raised alkaline phosphatase. Serum albumin was 30 g/L. Chest x-ray was normal.

Ultrasonography revealed marked dilatation of the intrahepatic biliary tree, and the patient was thought to have infestation with the liver fluke and concomitant cholangiocarcinoma (an association that we commonly see). At laparotomy a

Correspondence to: Dr. S. Ratanarapee, Department of Pathology, Faculty of Medicine, Siriraj Hospital, Mahidol University, Bangkok 10700, Thailand.

proximal ducts, Figure 1. On exploration of the common bile duct, irregularity and nodularity of the mucosal surface were observed. A few adjacent lymph nodes were enlarged but not adherent to the duct, and four were excised for biopsy. The appearances were still consistent with cholangiocarcinoma of the bile duct with probable nodal metastasis. A 3 cm ellipse was removed from the anterior wall of the common duct, and the duct was closed around a T-tube. Microscopic examination of the gall bladder showed non-specific chronic cholecystitis. The common bile duct showed typical tubercles composed of epithelioid cells and a few Langhans' giant cells with lymphocytes and fibrous tissue at the periphery, Figure 2. Acid-fast bacilli were clearly identified on Fite's stain. Similar lesions and organisms were identified in one of the lymph nodes. No malignant transformation of the epithelium was detected.

Postoperatively the patient received a full course of anti-tuberculous drugs (rifampicin, ethambutol and isoniazid). He made an uninterrupted recovery.

After 4 months the T-tube dislodged spontaneously from the duct and was removed, but liver function tests remained normal. Five years later he is in good health.

distended gall bladder was removed but contained no stones. The common bile duct was of firm-to-hard consistency with a nodular external surface. An intraoperative cholangiogram showed marked narrowing of its lumen with dilatation of the

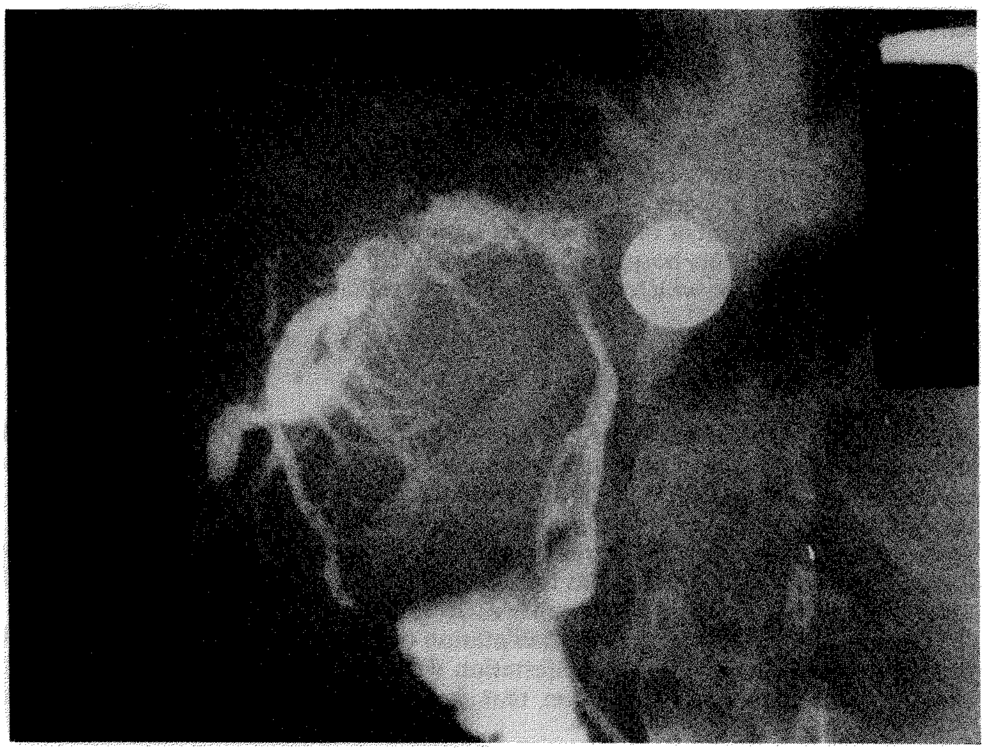

**Figure 1** Intraoperative cholangiogram showing marked narrowing of the common bile duct lumen and dilatation of the intrahepatic biliary tree.

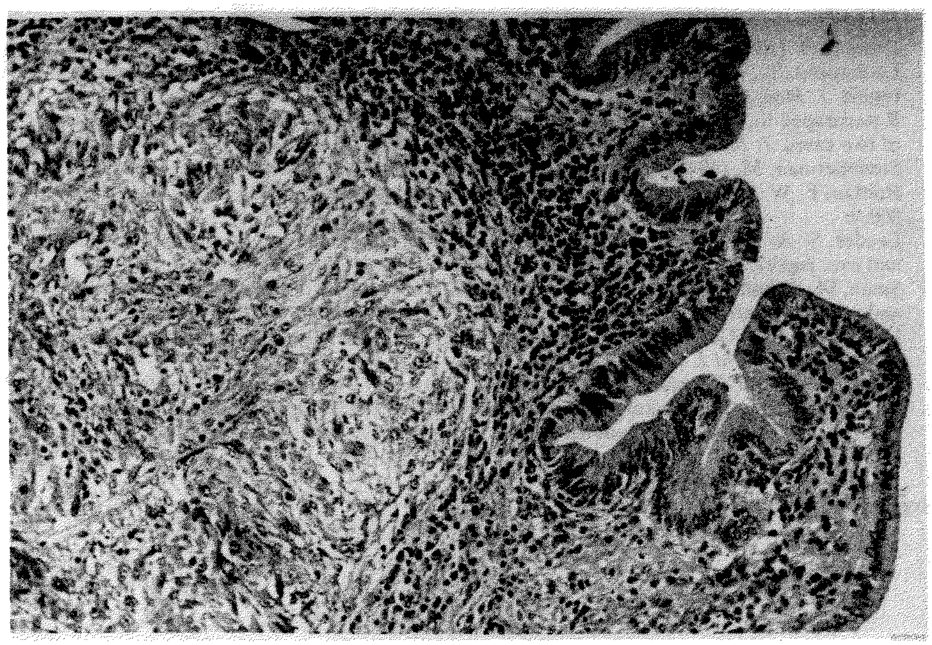

**Figure 2** Photomicrograph of the common bile duct showing a granulomatous lesion in the submucosa composed of many epithelioid cells with necrosis. Fibrous tissue and lymphocytes were seen at the periphery. (H & E, original × 100).

### DISCUSSION

The liver is usually affected in disseminated tuberculosis. There are three types of lesion: miliary (most often) then nodular and, least commonly, intrahepatic bile duct tuberculosis which produces characteristic cavities throughout the liver. In 1941, Stemmerman reported 45 cases of intrahepatic biliary tuberculosis, none of which involved the common bile duct[4]. Tuberculosis of the gall bladder is relatively rare[5,6], but again the common bile duct is spared. Since these organs have a similar epithelium and lymphatic drainage, the resistance of the common bile duct to tuberculosis is mysterious. Of similar rarity is tuberculous appendicitis, despite the prevalence of ileocaecal tuberculosis[7,8].

In the northeast and east of Thailand, liver fluke infestation (opisthorchiasis) is very common and cholangiocarcinoma is often associated[9,10]. Patients from this part of the country who present with obstructive jaundice are first suspected of having carcinoma of the intrahepatic bile duct, which may involve the common bile duct as well. Many patients only receive a palliative operation without the benefit of a tissue biopsy for pathological diagnosis.

The present case underlines the necessity for an accurate diagnosis on which to base possibly curative treatment.

### *References*

1. Dramas, S. (1981) Epidemiology of tuberculosis in Thailand. *Thai J. Tuberc. Chest Dis.* **2**, 53–4
2. Laohapand, T., Ratanarapee, S. and Chantarakul, N. *et al.* (1981) Tuberculous thyroiditis: a case report. *J. Med.Ass. Thailand* **64**, 256–60
3. Ratanarapee S., Bovornkitti, S. and Eungprabhant, V. (1985) Tuberculous myocarditis: a report of two cases. *J. Med. Ass. Thailand* **68**, 155–9
4. Stemmerman, M. (1941) Bile duct tuberculosis. *Quart. Bull. Sea View Hosp.* **6**, 316–24
5. Rankin, F. W. and Massie, F. M. (1926) Primary tuberculosis of the gall bladder. *Ann. Surg.* **83**, 800–6
6. Leader, S. A. (1952) Tuberculosis of the liver and gall bladder with abscess formation: a review and case report. *Ann. Intern. Med.* **37**, 594–606
7. Singh, M. K. and Arunabh, Kapoor V. K. (1987) Tuberculosis of the appendix a report of 17 cases and suggested aetiopathological classification. *Postgrad. Med. J.* **63**, 855–7
8. Homan, W. P., Grafe, W. R. and Dineen P. (1977) A 44-year experience with tuberculous enterocolitis. *World J. Surg.* **1**, 245–50
9. Sadun, E. H. (1955) Studies on Opisthorchis viverrini in Thailand. *Amer. J. Hyg.* **62**, 81–115
10. Koompirochana, C., Sonakul, D. and Chinda, K. *et al.* (1978) Opisthorchiasis: a clinicopathologic study of 154 autopsy cases. *Southeast Asian J. Trop. Med. Pub. Hlth.* **9**, 60–4

*(Accepted by S. Bengmark 2 July 1990)*

